# Detection of protein catalytic residues at high precision using local network properties

**DOI:** 10.1186/1471-2105-9-517

**Published:** 2008-12-04

**Authors:** Patrick Slama, Ioannis Filippis, Michael Lappe

**Affiliations:** 1 Structural Bioinformatics Group, Otto-Warburg Laboratory, Max Planck Institute for Molecular Genetics, Ihnestrasse 63-73, D-14195 Berlin, Germany

## Abstract

**Background:**

Identifying the active site of an enzyme is a crucial step in functional studies. While protein sequences and structures can be experimentally characterized, determining which residues build up an active site is not a straightforward process. In the present study a new method for the detection of protein active sites is introduced. This method uses local network descriptors derived from protein three-dimensional structures to determine whether a residue is part of an active site. It thus does not involve any sequence alignment or structure similarity to other proteins. A scoring function is elaborated over a set of more than 220 proteins having different structures and functions, in order to detect protein catalytic sites with a high precision, *i.e*. with a minimal rate of false positives.

**Results:**

The scoring function was based on the counts of first-neighbours on side-chain contacts, third-neighbours and residue type. Precision of the detection using this function was 28.1%, which represents a more than three-fold increase compared to combining closeness centrality with residue surface accessibility, a function which was proposed in recent years. The performance of the scoring function was also analysed into detail over a smaller set of eight proteins. For the detection of 'functional' residues, which were involved either directly in catalytic activity or in the binding of substrates, precision reached a value of 72.7% on this second set. These results suggested that our scoring function was effective at detecting not only catalytic residues, but also any residue that is part of the functional site of a protein.

**Conclusion:**

As having been validated on the majority of known structural families, this method should prove useful for the detection of active sites in any protein with unknown function, and for direct application to the design of site-directed mutagenesis experiments.

## Background

Determining the location of the active site of an enzyme is a crucial step in fundamental research as well as in drug design. In genetical studies, identifying mutations at or near an active site can help explain biological malfunctions. Knowledge of an active site, its geometry and physico-chemical properties, is essential for the efficient design of inhibitors of malignant proteins [1]. With extensive data now at hand on sequence and structure of genes and proteins, and broad functional knowledge, new methods aimed at determining the sequence and space location of unknown active sites from related or distant data have been elaborated over recent years. On specific protein families, such as DNA-binding proteins, methods analysing sequence only [2] or structural patterns [3,4] have proved efficient at detecting functional sites of such proteins. For more general applications, the distributions of different structural properties only [5] or in combination with physico-chemical properties of residues [6] have been studied. These properties were *e.g*. integrated into a neural network algorithm, in order to predict active site residues over various proteins with known structures [7]. A similar approach was used by Petrova, so as to predict active sites using Support Vector Machine on different structural and conservation properties of protein residues [8]. Another method, the 'Evolutionary Trace', relies on the hypothesis that important residues show slower mutation rates than non-functional residues in proteins and that, in three-dimensional structures, such residues are more likely to be clustered with each others than to be isolated in space [9-11]. Graph-derived approaches that detect the structural patterns of side-chain atoms that are recurrent over evolutionarily-related proteins were also proven to efficiently detect protein functional sites [12]. An optimal division of protein families into subfamilies, which followed the principles of phylogeny, enabled the identification of residues that were important for protein function [13]. Lastly, representation of protein structures as networks of interacting residues also enabled efficient detection of protein functional sites from three-dimensional structures [14-16].

This last representation, which facilitates mathematical manipulations of protein structures, is used in the current work. In such networks, each protein residue is a node, and two residues are connected by an edge if they have atoms within a given distance from each other. In the original definition, only contacts between amino-acids C_α _atoms were considered [17,18]. This description proved relevant for the detection of secondary structure motifs [19] and for comparing protein structures [20,21].

Closeness centrality of a node (a residue) within a network (a protein structure), as used in recent studies for the detection of protein catalytic sites [14,15], takes into account pathways that connect residues over the whole protein. Our belief was that interactions that take place at a local scale between residues would have a greater influence on the chemical and physical properties of residues than global properties. Non-bonding interactions have indeed very little chemical effect in the long range, as being due to electrostatic effects [22]. In addition, the modification of the electron richness of the side-chain atoms of a residue is in most cases not modified by residues that are distant from it by more than two non-covalent contacts.

The main features we thus focused on to describe protein residues were the number of 'local' neighbours of a node, i.e. nodes that are distant from this node by a path-length of one or two edges within the residue network. It has been shown that 2-connectivity, the count of the number of nodes distant by at most two edges from a given node, produced a similar efficacy at detecting protein active sites as closeness centrality [15]. Here we describe a combination of the counts of local neighbours, based both on all-atom contacts and side-chain atoms only contacts, with the distribution of residue types among protein catalytic sites. This score was tested for classifying residues as catalytic and non-catalytic using a set of over 220 proteins. Detection of catalytic sites was evaluated with respect to precision, or predictive value of positives, which reached a value superior to 28%. This performance is more than triple that of closeness centrality [14]. Our score also had highly improved performance using a measure that combined precision and coverage. Lastly, it was tested in detail over a set of eight proteins with different biological functions. Results suggested that our score was not only efficient at detecting only 'catalytic' residues, as defined in the Catalytic Site Atlas [23] but, more broadly, at detecting any residue involved in protein function.

## Results

### Detection of functional sites: general approach

Residue interaction networks were generated after the three-dimensional structures of a large test set of 226 proteins. Each of these proteins belonged to a distinct SCOP superfamily (see *Methods *for details) and had identified catalytic site residues, as being reported in the Catalytic Site Atlas [23]. This Atlas considers as catalytic the residues of a protein that are involved in catalytic reactions, under the following rules: being one of the reactant of the catalytic reaction, exerting an effect on a residue, a water molecule, a ligand or a cofactor which assists catalysis, stabilisation of a proposed transition state [6].

For each residue interaction network, different network parameters were analysed. Individual scores were next transformed into *MDev *values (see Additional file 1). *MDev *values do not involve standard deviations, and quantify deviations from average towards maximum for a given parameter (see *Methods *for definition).

As a benchmark to our method, prediction of protein catalytic sites was performed after the criteria defined by Amitai and Pietrokovski [14]. These criteria combine *Z*-score values on closeness centrality [24] and ranges for residue surface accessibility (RSA) values [25]. Using our set of 226 proteins, these criteria yielded a precision (see *Methods *for definition) of 8.22% for the detection of catalytic sites (Table 1).

**Table 1:** Comparison of the performance of predictions of catalytic residues using different scoring functions and threshold values on the extended protein set

	*F*_1_	*F*_2_	Precision	Coverage
Closeness + RSA^a^	15.13%	11.54%	8.22%	31.66%

Eq. 1^b^, *MDev1*	20.82%	-	15.42%	32.05%

Eq. 1, *MDev2*	-	20.56%	28.10%	9.91%

Calculations were run over the residue interaction networks derived from the 226 protein structures from our extended test set. Scoring functions used here are described in *Methods*. Values for precision and coverage were obtained over the whole set. *F*_1 _and *F*_2 _respectively represent the *F*-measure defined in *Methods *when using β = 1 and β = 2. ^a ^As proposed by Amitai [14]. RSA: residue surface accessibility. ^b^Residues were considered as catalytic if their *MDev *value for the scoring function defined in Equation 1 was superior to the indicated threshold value. Corresponding 'specificity' (equal to (p_-_, r_-_)/r_-_) values were 97.80% at *MDev1*, 99.68% at *MDev2*, and 95.57% when using closeness combined to RSA.

### Detection of catalytic sites: performance of our scoring function

Our scoring function combined three characteristics of a given residue: the number of residues in contact with it through side-chain atoms (*Dg*1_*SC*_), the number of residues located at a path-length of three (*Dg*3) and the type of the residue (Equation 1). It was used to detect catalytic residues over a set of 226 proteins belonging to different structural families. The score obtained for each residue was then transformed into a normalised *MDev *value. Moreover, the threshold value of *MDev *was optimized in order to produce a maximal value for a measure of performance that combined the precision and coverage values of the detection. Indeed, in order to have an efficient tool for the prediction of residues interesting for site-directed mutagenesis, it is important both to predict few non-catalytic residues (high precision) and to have a high likelihood that a catalytic site is effectively predicted as such (high coverage). Still, precision tends to increase with increasing values of thresholds, while coverage displays an opposite trend. We thus optimised our detection of catalytic sites for a maximal value of a measure of performance which combined precision and coverage, the *F*-measure [26]:

$$F_{\beta }=\frac{(1+\beta^{2})\times (\text{precision }\times \text{coverage)}}{\beta^{2}\times \text{coverage }+\text{precision}}.$$

**Figure 1:**
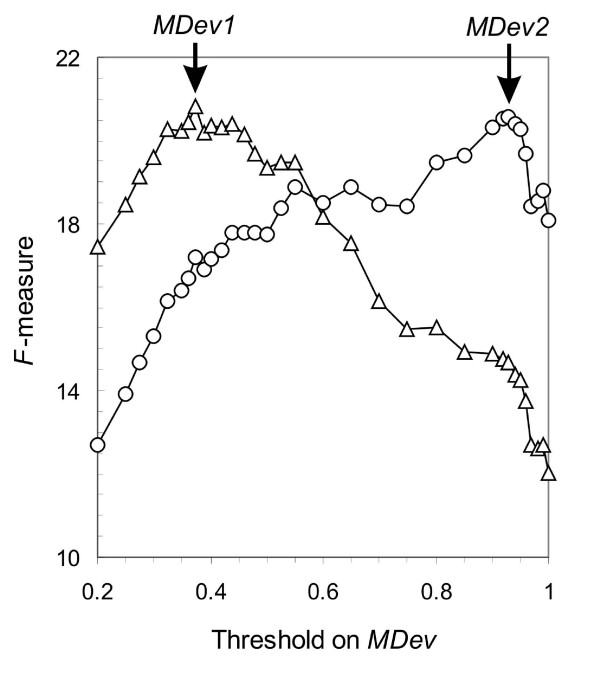
**Values of *F-measure *as a function of threshold on *MDev *for scores obtained using *Equation 1*.** Scores were calculated for all residues from the extended test set. The values of the effectiveness measures *F*_1 _and *F*_2_, as defined in *Methods *(with β = 1, as triangles, *F*_1_, and with β = 2, *F*_2_, as circles), were calculated when classifying as catalytic the residues with an *MDev *value superior to thresholds ranging from 0.2 to 1. The respective thresholds that produced maximal values for respectively *F*_1 _and *F*_2_, *MDev1 *and *MDev2*, are indicated.

This measure of effectiveness was maximised for the extended set of 226 proteins in two conditions: at *β *= 1, *i.e*. when precision and coverage were given a similar importance, and at *β *= 2, with increased importance on precision. The maximum values for *F*_1 _and *F*_2 _when using our scoring function were respectively 20.82% and 20.56%, with corresponding threshold values of *MDev1 *= 0.375 and *MDev2 *= 0.93 (Figure 1). The corresponding values for precision and coverage are displayed in Table 1.

When comparing these values with those obtained using closeness centrality and RSA, our scoring function produced a two-fold increase in precision when using *MDev1 *and more than three-fold increase in the *MDev2*, with calculations performed on the same data set (Table 1). Moreover, the overall performance was improved with respect to that same method, both when using *F*_1 _at threshold *MDev1 *and *F*_2 _at threshold *MDev2 *(Table 1).

**Figure 2:**
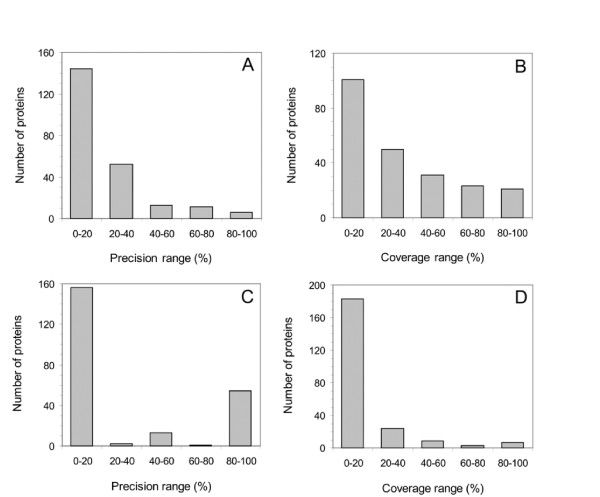
**Per-protein ranges obtained on the precision (A and C) and coverage (B and D) of the detection when considering as catalytic the residues with an *MDev *superior to *MDev1 *(A and B) or to *MDev2 *(C and D). ***MDev *values were calculated on each residue of the extended test set from scores calculated according to *Equation 1*.

Distributions of per-protein performance values were homogeneous at threshold *MDev1*, while precision values were split between low and high values at *MDev2 *(Figure 2). The average of the per-protein coverage was 32.0% when considering as catalytic the residues with an *MDev *value superior to *MDev1*, with 75 of the 226 proteins having a coverage value above 40%. When using threshold *MDev2*, the average of the per-protein precision was 27.3%, with a quarter of the 226 proteins having a precision greater than 80% (Figure 2).

### Validation set: detailed performance and re-consideration of 'catalytic' residues

In order to evaluate the quality of our detection method at a structural and functional level, eight proteins belonging to different functional families were analysed into more detail. These proteins along with their catalytic sites and the residues predicted as catalytic by our scoring function are presented in Table 2.

**Table 2:** Results at the residue scale: detection of catalytic and functional residues over the proteins from the validation set.

Protein	Residues predicted as catalytic^a^	Non-detected catalytic residues	Comments^b^
TEM β-lactamase	Lys73*, Glu166, Asp233, **Lys234**	Ser70, Ser130	Lys234 forms H-bond with substrate analogue-binding water

Pancreatic phospholipase	Arg6, **Glu46**, **His48**, Asp49*, Asp99*	Gly30	Asp49 binds Ca

Alkylguanine-transferase	Tyr69, His71, His146*, Arg147, Tyr158, Lys165, Glu172*	Asn137, Cys145	Glu172→His146 activates Cys145 by deprotonation, Lys165 mutations affect activity

Ubiquitin-conjugating enzyme 1	Lys36, **Asp55**, Asp72	Cys88	Detected residues define a single site in structure

Phenylalanine hydroxylase	His138*, Asp139, His143*, Glu184*	Ser203	Asp139 forms H-bond with Fe-bound H_2_O

Prolyl-isomerase 1	His59*, Glu145, His157*	Cys113	Glu145 plays a role in the two-domain arrangement of the protein

Ferric binding protein	His9*, Glu57*, **Arg101**, Arg103, Glu144, Glu264	Tyr195, Tyr196	Arg101 (not conserved) interacts with ligand, Glu57 interacts with ligand and binds iron

Bovine β-trypsin	His40, Asp189, Ser190, **Asp194**, Tyr228, Lys230	His57, Asp102, Gly193, Gly196, Ser214	Asp189 forms H-bond with substrate-bound water, Tyr228 is H-bonded to Asp189 through H_2_O

^a ^All residues predicted using our scoring parameter at threshold *MDev1*. In bold, those also predicted at threshold *MDev2*. With * superscript, residues that are 'catalytic' according to CSA definition. Residues that are 'functional' according to our definition (see text) but not 'catalytic' according to CSA are underlined. ^b^Derived from the analysis of multiple crystallized states. See text for references.

For this smaller set we considered 'catalytic' residues as well as 'functional' ones based on extensive analysis of existing literature on each of these proteins. 'Functional' residues, as opposed to the more restrictive definition of 'catalytic' residues of the CSA, included all residues which had a proven role either in the binding of substrate(s) or cofactor(s), as well as in the catalytic activity of the protein, even though not directly involved in the catalytic reaction.

Detection of 'catalytic' residues was run on the two threshold values which yielded maximal values for *F*_1 _and *F*_2_, *MDev1 *and *MDev2 *respectively, with results summarized in Table 2 and Table 3. As the second criterion is more restrictive than the first one, all residues predicted as positives using the second threshold were also predicted as positives using the first one.

**Table 3:** Comparison of performances of detections carried out on the validation set using different threshold values

	Catalytic			Functional
Threshold on *MDev*	*F*-measure^a^	Coverage	Precision	Precision

*MDev1*	33.5%	44.4%	31.6%	65.8%
*MDev2*	27.8%	20%	45.5%	72.7%

Calculations were run on the 8 protein structures of the validation set. Our scoring function (*Equation 1*) was used, with detection at two different thresholds. Residues were considered as positives (catalytic or functional) if their *MDev *value was superior to the threshold value. Performances are expressed with respect to the whole set. ^a^Values correspond to measure *F*_1 _for *MDev1 *and *F*_2 _for *MDev2*.

TEM β-lactamase is responsible for bacterial resistance to penicillins and cephalosporins antibiotics. For this protein, catalytic Ser70 was not detected, while the two residues which are likely to play the role of a base for the activation of this serine, Lys73 and Glu166 [27-29], were detected (Table 2). Asp233 is strictly conserved over known class A β-lactamases [30].

In pancreatic phospholipase, an enzyme involved in the metabolism of phospholipids, catalytic Asp99 was detected, but only at *MDev1*. Active-site His48, calcium-binding Asp49 and substrate-binding Arg6 [31,32] were also detected.

Alkylguanine transferase is a key enzyme in DNA repair which catalyses the dealkylation of O6 from guanine nucleotides. Prediction on this enzyme yielded numerous positive residues, among which catalytic Cys145 was not present. Still, the two residues proposed as activating this residue by deprotonation, His146 and Glu172 [33], were predicted as catalytic. All but two of the remaining residues predicted as catalytic had either a structural role in the arrangement of the active site (Tyr158) or a functional role (Arg147, Lys165) [34,35].

**Figure 3:**
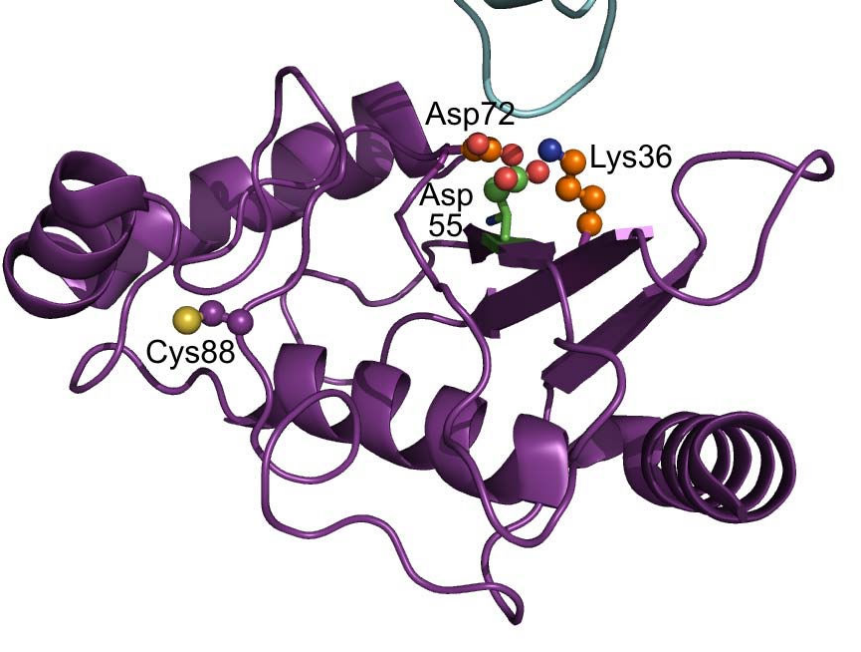
**Catalytic residues detected on ubiquitin-conjugating enzyme 1 using our scoring function.** Side-chains of residues predicted as catalytic at thresholds *MDev1 *and *MDev2 *are shown as ball-and-sticks, with carbon atoms in orange and green, respectively. The second monomer present in the crystal structure is shown in cyan. The active-site cystein residue, Cys88, is shown as ball-and-sticks, with carbon atoms in purple.

For ubiquitin-conjugating enzyme 1, an enzyme involved in the transfer of ubiquitin entities to protein substrates, none of the three residues predicted as catalytic possessed a described role in enzyme activity [36]. Still, these residues defined a single pocket in the crystal structure, which is located at the surface of the protein, and facing the second monomer present in the structure (Figure 3). These residues could thus play a role in interactions of ubiquitin-conjugating enzyme with other proteins.

**Figure 4:**
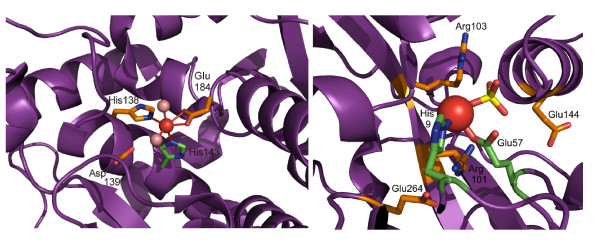
**Visualisation of the catalytic and functional residues detected using our scoring function on two proteins from the validation set.** Side-chains of residues predicted as catalytic (see Table 2) when using thresholds *MDev1 *and *MDev2 *are shown as sticks, with carbon atoms in green and orange, respectively. Bonds to iron are shown as solid lines. Left: Phenylalanine hydroxylase. Iron is shown as a red sphere and water in pink. Right: ferric-binding protein. Iron is shown as a red sphere and phosphorous in yellow.

**Figure 5:**
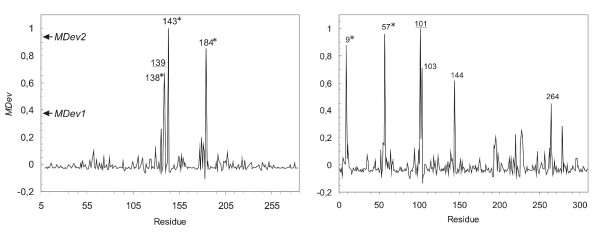
**Distribution of *MDev *values for our scoring parameter as obtained for phenylalanine hydroxylase (left) and ferric-binding protein (right).** Residue numbers correspond to the numbering of the PDB structure and are indicated for residues with values of *MDev *on our scoring function superior to *MDev1*. Catalytic residues are indicated with a * superscript, functional residues are underlined.

Phenylalanine hydroxylase catalyzes the aromatic-ring hydroxylation of amino-acid phenylalanine to produce tyrosine. Three of the ligands of the active-site iron of this enzyme were detected (Table 2), while the last detected residue (Asp139) is hydrogen-bonded to an iron-binding water molecule [37] (Figure 4). It is interesting to note that, in spite of the length of the protein sequence (275 residues in crystallised structure), all residues detected play a functional role. A distribution of the *MDev *values for our scoring function on each residue of this protein is shown in Figure 5.

Prolyl-isomerase 1 catalyses the cis-trans isomerisation of proline residues, and recent studies have linked this protein to cancer and Alzheimer's disease [38]. His59 and His157 (Table 2) are catalytic residues that are located at the bottom of the pocket for substrate interaction (Figure 4), while Glu145 is located in the region that links the isomerase domain to the WW domain of the protein [39,40].

In the Fe^3+^-binding protein, a protein involved in bacterial iron uptake, one residue detected at *MDev2 *is an iron ligand (Glu57), and the second one (Arg101) interacts with substrate phosphate and is located close to iron (~4 Å for terminal nitrogens, Figure 4). Among the four additional residues predicted at *MDev1 *(Figure 5), one is an iron ligand (His9) and one (Glu264) hydrogen binds both Arg101 and a water molecule close to the active-site iron.

None of the residue predicted by our method in protease bovine β-trypsin is involved in catalytic function (CSA definition, Table 2). Still, as observed in more recent inhibitor-bound structure [41], four of the six residues predicted as catalytic are directly hydrogen-bonded to inhibitor molecule (Ser190), or to water molecules that are present at the active site. Moreover, while Asp194, which was detected at *MDev2*, is not involved in docking, its two sequence neighbours, Gly193 and Ser195, directly interact with bound inhibitor and are labelled as catalytic residues in the PDB structure.

Overall, for six out of the eight proteins, catalytic residues were detected using our scoring function, though with many positive residues that were not catalytic according to CSA definition (Table 2). Still, for all but one of the proteins, all residues predicted at *MDev2 *corresponded to 'functional' residues. We labelled as such the residues directly involved in catalytic reactions or in substrate binding, as well as those located near an active site that had a proven influence on catalytic rate, as deduced from experimental results. When using either *MDev1 *or *MDev2 *threshold value, the precisions obtained were higher than those obtained on the extended set (Table 1 and Table 3). Coverage values of catalytic residues were respectively of 19.2% and 46.2% at *MDev1 *and *MDev2*, to be compared to 9.9% and 32.1% respectively on the extended set (Table 1). As for 'functional' residues, precisions obtained were 72.7% and 65.8% respectively at *MDev1 *and *MDev2 *(Table 2 and Table 3). Even though precision values between this restricted set and the extended set cannot be compared due to the small size of the former, increase in precision when detecting 'functional' residues as opposed to 'catalytic' residues only (Table 3) is highly interesting for the biological relevance of our detection method. Distribution of *MDev *values showed that this measure was efficient at discriminating between residues, with very few non-functional residues at high *MDev *values, as observed *e.g*. on phenylalanine hydroxylase and the Fe^3+^-binding protein (Figure 5).

## Discussion

The present study proposes a new method for the prediction of catalytic sites in proteins based on their residue-residue contact networks. This method only relies on the knowledge of protein three-dimensional structures, with no requirement of functional attribution or sequence alignment to other proteins, and can thus directly be applied to proteins with no known homologues.

### Definition of residue-residue contacts and local network parameters

Residue interaction networks were built from protein three-dimensional structures using all non-hydrogen atoms to define contacts between residues. Edges were distinguished on whether the atoms involved belonged to the side-chain or backbone of each residue. This distinction proved relevant, since *Dg*1_*SC *_(defined in *Methods*) both produced a higher average *MDev *value over catalytic residues from the extended set and was less correlated to *Dg*3 than *e.g. Dg*1 (see Additional file 2).

Our results therefore prove that the use of these local (*Dg*1_*SC*_) and semi-local (*Dg*3) parameters within the residue-interaction network that describes a protein structure enabled a better detection of protein catalytic sites than closeness centrality, a parameter that considers path lengths between all residues of the network. They therefore suggest that local or semi-local organisation of residues is more critical than whole-protein structural information to define them as catalytic or not, as shown by the increased precision of detection obtained over 226 representative protein structures (Table 1). They moreover validate our initial hypotheses of a stronger relevance of chemically significant residue-residue contacts to define catalytic sites.

It is likely that an even better detection shall be achieved in the future by using different types of local network parameters, possibly by combining them to other physico-chemical properties. Still, it is to be noted that combination of our two network parameters to the crystallographic B-factor for each residue did not produce a higher precision of the detection than that obtained with the scoring function of Equation 1 (data not shown).

### Choice of binary descriptors

The final performance of the detection was measured using both precision (predictive value of positives) and coverage, instead of the more classical specificity and sensitivity (coverage) combination. This choice was motivated by two reasons: a practical one and a methodological one. The practical reason is the applicability of the method to the choice of protein amino-acids that would be interesting for site-directed mutagenesis experiments. Both a high rate of correctly predicted sites, *i.e*. a low false detection rate, and a high coverage of functional sites, are the characteristics one would require for efficient prediction. Indeed, these two criteria will provide both a low rate of negative experiments and a high likelihood of detecting the active site for a given protein. The methodological reason has grounds in the rates of occurences of catalytic residues in the extended set. The 226 proteins have 62083 amino-acids in total, with 777 catalytic residues. Therefore, the sample is highly unbalanced, with percentages of real positives (*r*_+_) and real negatives (*r*_-_) over this set of respectively 1.3% and 98.7% of full sample. In such a case, small variations in the number of correct predictions (and therefore of non-correct predictions) will have a low influence on measures of performance that use ratio to the number of residues predicted as non-catalytic (*p*_-_), *e.g*. specificity or true negative rate. On the contrary, similar variations will have a high influence over coverage or predictive value of positives, whose evaluation only involves positive residues (predicted or real). For these two reasons, precision and coverage were chosen as performance measures.

In order to obtain a single measure of performance for our detection, precision and coverage were combined into an effectiveness measure, the *F*-measure (see *Methods*). Thresholds on *MDev *that produced maximal values for this effectiveness measure were chosen in two conditions: one where an equal relative importance was conferred to precision and coverage (β = 1, threshold *MDev1*), and one where precision was given a more important weight (β = 2, threshold *MDev2*). The use of two distinct threshold values provides the user with two sets of residues to analyse of different sizes: a broad set presenting a high coverage, with low chances to miss an active site and more experiments to perform (*MDev1*), and a narrow set, with both fewer false positives and lower chances to hit an active site, and also fewer experiments to perform (*MDev2*).

### Comparison to other methods

Table 1 compares the performance of our method with one that combines closeness centrality and RSA [14], using our extended set of 226 proteins. When using the combination of *Z*-score on closeness centrality and RSA criteria as proposed by Amitai *et al*. [14], a value of 8.2% was obtained for the precision of the detection of catalytic sites (Table 1). This value is to be compared with that of 28.1% obtained using our method (Table 1). As for the *F*-measure, respective values of 15.1% and 11.5% were obtained when applying the method of Amitai and coworkers at β = 1 and β = 2, while our method produced values superior to 20% (Table 1).

The final performance of our method was also compared to that proposed by Petrova, which uses Support Vector Machine over 7 residue attributes. In that study, predictive accuracy $\left(\frac{\left(p_{+},r_{+})+(p_{-},r_{-}\right)}{r_{+}+r_{-}}\right)$ is used as a performance measure, for which an optimum value of 87% was obtained [8], while a similar calculation on our method yielded a value of 98.6% over our extended test set.

Lavery and co-workers used calculations of propensity of protein residues to be locally displaced, or mechanical rigidity, as a tool for detecting catalytic sites over various proteins [42]. Their method produced both a high specificity and coverage of predictions, with respective values of 74% and 78% over 100 proteins. These values correspond to a precision of 3.3% for the detection of catalytic sites. It can also be noted that this method involved time-costing molecular mechanics calculations on each protein structure, in comparison to ours which could be run in a few seconds on each protein.

Further comparison to our method can also be performed using the receiver-operator characteristic (ROC) curve (see Additional file 3) though, as discussed earlier, specificity is not a relevant performance measure for such unbalanced samples.

### 'Catalytic' vs. 'functional' residues

When comparing the performance of the method over the extended set and the restricted set, it was observed that a higher precision was obtained on the second one than on the first. One trivial bias could be due to the much smaller size of the validation set, with 8 proteins *vs*. 226. Still, an important difference in the measure of precision over the validation set has to be noted. In the Catalytic Site Atlas, residues are labelled as 'catalytic' if they are involved in the catalytic reaction in the strict sense [6]. However, when testing the performance of our scoring parameter on the validation set, we manually defined residues that were important for activity on grounds of functional and structural experiments. One difference between the two definitions is, for instance, that residues that bind an active-site metal ion or are involved in substrate docking are considered as functional, in our definition, but are not present in the Atlas. We could thus observe that our scoring function, while optimised for detecting 'catalytic' residues from the Catalytic Site Atlas, produced a higher precision at detecting 'functional' residues, both at *MDev1 *(72.7% *vs*. 45.5%) and *MDev2 *(65.8% *vs*. 31.6%), than at detecting purely catalytic sites (Table 3). It should also be noted that definition of functional residues as used for the validation set, which originates from literature searches for each protein, is likely to be more accurate than that used to define 'catalytic' sites in the CSA. Indeed, a majority of the residues listed in the CSA are defined as catalytic using only information from analogous proteins and sequence comparison methods [23]. These results thus further validate the current method as a solid one for detecting functional residues present at enzyme active sites, which can play crucial roles in enzyme activities [35], and not only residues directly involved in catalytic reactions.

## Conclusion

A scoring function based on residue local network descriptors, which did not involve any sequence alignment of the proteins under study or any attribution of function to proteins, was calculated for each residue of a set of proteins with known active sites. Residues were labelled as catalytic when their resulting score was superior to a given threshold value, and the threshold was fitted in order to obtain a minimal false detection rate, or maximal precision. Our detection method produced a precision of 28.1% for the catalytic sites of 226 proteins with variable folds and function, a more than three-fold increase compared to existing methods (8.2% for closeness centrality combined with residue surface accessibility). On a smaller set of 8 proteins, use of the same method produced a precision of 45.5% for the detection of catalytic sites and, when extending the measure of performance to all residues that were crucial to protein activity, which we coined 'functional', precision of the detection increased to 72.7%. The present scoring function, while optimised for 'catalytic' residues, thus proved even more efficient at detecting 'functional' residues. The high precision obtained with this method proved the influence of the local environment of residues in structurally organising protein active sites. The method should be of help in designing site-directed mutagenesis experiments with a low time-cost.

### Availability

The method can be applied to any protein structure (X-ray, NMR or model) by submission of a PDB file to the corresponding author. Two sets of residues will be produced: one that will only consider the residues predicted as catalytic or functional at high coverage and average precision (*MDev *= *MDev1*), and another set, which will be a subset of the previous one, with the residues predicted at high precision and average coverage (*MDev *≥ *MDev2*). An online version for direct submission will soon be available on our web-page .

## Methods

### Definition of the extended test set

A non-redundant set of enzymes was selected from the Catalytic Site Atlas  as in version 2.2.1. Proteins present in this Atlas were mapped with the Structural Classification Of Proteins (SCOP, ). Superfamilies which included fewer than two proteins, as well as those belonging to the 'low resolution proteins' and to the 'designed proteins' classes, were excluded. A single protein was randomly selected for each remaining SCOP superfamily. The resulting set contained 226 proteins as listed in additional file 4, with 62803 amino-acids, among which 777 labelled as 'catalytic' in the Catalytic Site Atlas.

### Networks of residue interactions

Residue interaction networks were calculated from protein three-dimensional structures on all atom-to-all atom contacts. Two residues were considered in contact if they had a pair of not covalently-connected atoms that laid within a distance of 4.2Å. Side-chain-to-side-chain contacts represented contacts between any two atoms not belonging to the amino-acid moiety (C_α_, N or carbonyl group) of two distinct residues.

Different network parameters were calculated for each residue within the resulting networks, such as direct neighbours defined on all-atom contacts (*Dg*1) or on contacts involving only side-chain atoms (*Dg*1_*SC*_). More generally, the *Dg*p value for a given node, with p an integer number, represents the number of nodes that are located at exactly p steps (or edges) from that node.

Standard values of *Dg*1_*SC *_per residue type were calculated for a set of proteins that was obtained from the Pisces Protein Sequence Culling Server . Networks of residue interactions were calculated for 1858 proteins with less than 25% sequence identity and resolution better than 1.8Å. The resulting averaged *Dg*1_*SC *_value for each amino-acid type was referred to as <*Dg*1_*SC*_>. Parameter *Dg*1_*SC *_was thus transformed into $Dg1_{SC-R}=\frac{Dg1_{SC}}{<Dg1_{SC}>}$. Similar calculations were performed on *Dg*1 for all residues from this set and enabled us to define a <*Dg*1> value for each amino-acid type. This normalised value was preferred to *Dg*1_*SC*_, since the number of direct neighbours was highly dependent on the residue type, an effect which is not observed in *Dg*1_*SC*-*R*_. In contrast, the influence of the residue type on *Dg*2 or *Dg*3 was smaller, so no normalisation was used on these parameters.

Closeness centrality for a node within a given network was defined as the inverse of the average shortest path-length to all other nodes, as used by Amitai [14].

### Statistical analysis

For each network scoring function (*x*) used to characterize a residue, the average ($\bar{x}$), maximum (*x*_max_) and standard deviation (*σ(x)*) for that score over each protein residue-residue contact network were calculated. Parameters were then classified either on *Z*-scores: $Z-\text{score}=\frac{x-\bar{x}}{\sigma (x)}$ or on $MDev=\frac{x-\bar{x}}{x_{\max}-\bar{x}}$. *MDev *was chosen in order to measure a deviation from maximum, rather than a deviation from the average as in standardised *Z*-score. It was moreover preferred to a plain ranking with selection of a fixed number of residues for all proteins, since the number of residues that define an active site can differ from a protein to another and between catalytic functions. *MDev *produced a value of 0 for a residue with a parameter value *x *equal to its average over the protein it belonged to, 1 for the residue(s) with *x *equal to *x*_max _for the protein, and negative values for residues with *x *values lower than the average parameter value over the protein.

Residues were considered as 'positives', *i.e*. predicted as belonging to a catalytic site, if their *Z *or *MDev *value was superior to a given threshold value for the score under consideration. Each score was finally evaluated with respect to precision (or predictive value of positives, ratio of correct prediction of positives over all prediction of positives) and coverage of positives (ratio of the number of correctly predicted catalytic residues over the number of residues that were effectively catalytic) over the protein set. With *r*_+ _and *r*_- _the number of real catalytic and non-catalytic residues in the set under consideration, *p*_+ _and *p*_- _the number of protein residues respectively predicted as involved and not involved in catalysis, (*p*_+_, *r*_+_) the number of correctly predicted catalytic residues, the values for the different measures were:

$$\begin{array}{cc} \text{precision}=\frac{\left(p_{+},r_{+}\right)}{p_{+}}, & \text{coverage}=\frac{\left(p_{+},r_{+}\right)}{r_{+}}. \end{array}$$

For measuring the performance of the detection method, a combination of precision and coverage was also used: the *F*-measure [26], $F_{\beta }=\frac{(1+\beta^{2})\times (\text{precision }\times \text{coverage)}}{\beta^{2}\times \text{coverage }+\text{precision}}$.

### Elaboration of the scoring function

The scoring function we derived from network parameters was defined using a combination of two network parameters with a residue-type frequency. Use of two network parameters was justified by the fact that any single parameter considered produced poor predictive values. The parameters used were *Dg*3 and *Dg*1_*SC*-*R*_, because *i) *they displayed a distribution of *MDev *values biased towards 1 for catalytic residues from the extended set and *ii) *they possessed the smallest pairwise correlations between the parameters that were considered (see Additional file 2).

The likelihood of each amino-acid to be a catalytic residue was considered in our scoring function. A subset of the Catalytic Site Atlas with no overlap with the extended test set was defined, with the following rules: only entries with literature evidences were included, a single chain was considered for PDB entries with multiple chains present in the Atlas, and proteins from the 'low resolution proteins' and 'designed proteins' classes were excluded. The resulting set included 546 proteins, for a total of 1478 catalytic residues. Each residue was thus attributed a *D*_*type *_value, which represented the percentage of residues of this given type (Ala, Asp, Cys...) present over these 1478 catalytic residues.

The combined scoring function attributed to each residue the following score:

(1) $$Dg3^{3}\times \exp\left(k_{exp}\frac{Dg1_{SC}}{<Dg1_{SC}>}\right)\times \left[1+k_{\text{type}}\times \left(D_{type}-med\left[D_{type}\right]\right)\right]$$

Variable parameters *k*_*exp *_and *k*_*type *_were chosen in order to produce a maximal performance value for the detection, and had final values of 0.25 and 50, respectively.

### Validation set

For a validation at the residue scale of the scoring parameter defined on the extended set, eight proteins belonging to different functional classes were chosen for detailed analysis. These proteins were as follows, with respective PDB three-dimensional structures used to generate the residue contact networks: TEM β-lactamase from *Esch. coli *(E.C. 3.5.2.6, PDB entry 1m40, adduct with transition-state analogue boronate), porcine pancreatic phospholipase (E.C. 3.1.1.4, PDB entry 1p2p, calcium bound), DNA-alkylguanine transferase (E.C. 2.1.1.63, PDB entry 1eh6, unbound form), ubiquitin-conjugating enzyme 1 from *Sacch. cerevisiae *(E.C. 6.3.2.19, PDB entry 1fzy, unbound form), phenylalanine hydroxylase from *Chr. violaceum *(E.C. 1.14.16.1, PDB entry 1ltv, iron cofactor bound), human peptidyl-prolyl isomerase (E.C. 5.2.1.8, PDB entry 1pin, substrate Ala-Pro bound), ferric binding protein from *Hæm. influenzae *(PDB entry 1mrp, iron and phosphate bound) and bovine β-trypsin (E.C. 3.4.21.4, PDB entry 5ptp, calcium bound).

## Authors' contributions

ML provided funding and working tools to IF and PS. IF contributed to the creation of the computer codes and performed part of the calculations. PS created and analysed the extended and validation sets and created and optimised the scoring function. PS and IF wrote the manuscript.

## Supplementary Material

Additional file 1 **Distribution of *MDev *values calculated on different network parameters over the catalytic residues present in the extended set of proteins.** The figure presents distribution of *MDev *values for the different network parameters that were considered, in order to evidence biases towards the maximum *MDev *value of 1. Click here for file

Additional file 2 **Pairwise correlations for different network parameters for the catalytic residues present in the extended set of proteins.** Correlation values for different network parameters over the residues labelled as 'catalytic' in the Catalytic Site Atlas are given for the 226 proteins from the extended set of proteins. Parameters used are closeness centrality, used as a benchmark, and neighbour counts *Dg*1, *Dg*2 and *Dg*3, as well as normalised count *Dg*1_*SC*-*R*_. Click here for file

Additional file 3 **Receiver-operator characteristic curve for the detection of catalytic sites over the extended set of proteins when using the scoring function defined in *Equation 1*.** The curve shows the relationships between specificity and coverage when using our scoring function for the detection catalytic sites. Each point corresponds to a different threshold on *MDev *values. Click here for file

Additional file 4 **Description of the extended set of proteins.** PDB identity and chain for all proteins from the extended set are provided, as well as the corresponding SCOP domain of the chain used. Click here for file
