# Supplementary material for: Detection of protein catalytic residues at high precision using local network properties
Source: BMC Bioinformatics. 2008 Dec 4;9:517. doi: 10.1186/1471-2105-9-517 (PMC2632678; doi:10.1186/1471-2105-9-517)
Supplement: Additional file 1 — Distribution of MDev values calculated on different network parameters over the catalytic residues present in the extended set of proteins. The figure presents distribution of MDev values for the different network parameters that were considered, in order to evidence biases towards the maximum MDev value of 1. [file 1471-2105-9-517-S1.doc]

**Additional file 1.** Distribution of MDev values calculated on different network parameters over catalytic residues. *Dg*1*_SC_* (striped bars), *Dg*1*_SC-R_* (white bars), *Dg*2 (grey bars) and *Dg*3 (dark grey bars) *MDev* values for the residues labelled as catalytic over the 226 proteins from the training set. An *MDev* value of 0 corresponds to a parameter value equal to the average value over the protein to which the given residue belongs.
