# Supplementary material for: Detection of protein catalytic residues at high precision using local network properties
Source: BMC Bioinformatics. 2008 Dec 4;9:517. doi: 10.1186/1471-2105-9-517 (PMC2632678; doi:10.1186/1471-2105-9-517)
Supplement: Additional file 2 — Pairwise correlations for different network parameters for the catalytic residues present in the extended set of proteins. Correlation values for different network parameters over the residues labelled as 'catalytic' in the Catalytic Site Atlas are given for the 226 proteins from the extended set of proteins. Parameters used are closeness centrality, used as a benchmark, and neighbour counts Dg1, Dg2 and Dg3, as well as normalised count Dg1SC-R. [file 1471-2105-9-517-S2.pdf]

Additional file 2. Pairwise correlations for different network parameters for the catalytic residues present in the extended set of proteins.

	Closeness centrality	*Dg*2	*Dg*3
Closeness centrality	1	0.641	0.661
*Dg*1	0.490	0.724	0.505
$Dg1_{SC-R}$	0.332	0.526	0.377
