# Supplementary material for: Detection of protein catalytic residues at high precision using local network properties
Source: BMC Bioinformatics. 2008 Dec 4;9:517. doi: 10.1186/1471-2105-9-517 (PMC2632678; doi:10.1186/1471-2105-9-517)

Additional file 3. Receiver-operator characteristic (ROC) curve for the detection of catalytic sites over the extended set of proteins when using the score defined in *Equation 1*. *MDev* values used range from -0.25 (Coverage= 100%) to 1 (Specificity= 99.74 %).

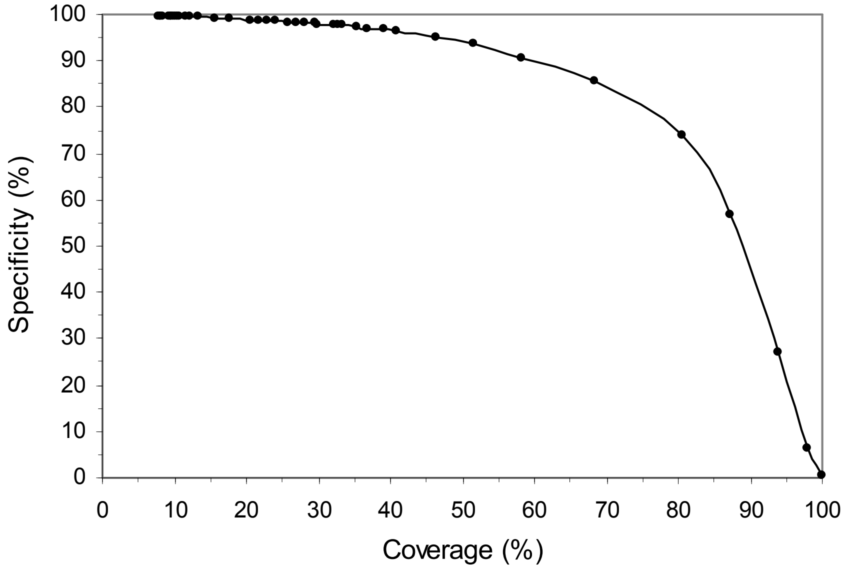

Supplement: Additional file 3 — Receiver-operator characteristic curve for the detection of catalytic sites over the extended set of proteins when using the scoring function defined in Equation 1. The curve shows the relationships between specificity and coverage when using our scoring function for the detection catalytic sites. Each point corresponds to a different threshold on MDev values. [file 1471-2105-9-517-S3.pdf]
