# Supplementary material for: Detection of protein catalytic residues at high precision using local network properties
Source: BMC Bioinformatics. 2008 Dec 4;9:517. doi: 10.1186/1471-2105-9-517 (PMC2632678; doi:10.1186/1471-2105-9-517)
Supplement: Additional file 4 — Description of the extended set of proteins. PDB identity and chain for all proteins from the extended set are provided, as well as the corresponding SCOP domain of the chain used. [file 1471-2105-9-517-S4.doc]

**Additional file 4**. Description of the extended set of proteins. The PDB entry and chain used is given for each of the 226 proteins, as well as its corresponding SCOP (Structural Classification Of Proteins) domain. Each SCOP superfamily is represented by at most one protein chain.

| PDB:chain | SCOP | PDB:chain | SCOP |
| --- | --- | --- | --- |
| 1ksc:A | a.102.1.2 | 1jf8:A | c.44.1.1 |
| 1kkt:A | a.102.2.1 | 1t4j:A | c.45.1.2 |
| 1hv6:A | a.102.3.1 | 1xwc:A | c.47.1.1 |
| 1tnu:J | a.102.4.3 | 1cfr:A | c.52.1.7 |
| 1r76:A | a.102.5.1 | 1t75:A | c.53.2.1 |
| 1ixe:A | a.103.1.1 | 1wsj:C | c.55.3.1 |
| 1gjm:A | a.104.1.1 | 1pw7:A | c.56.2.1 |
| 1iw8:F | a.111.1.1 | 1ryn:A | c.56.3.1 |
| 1mzc:A | a.118.6.1 | 1a2z:D | c.56.4.1 |
| 1w3b:A | a.118.8.1 | 1bav:B | c.56.5.1 |
| 1p6d:A | a.124.1.1 | 1bou:D | c.56.6.1 |
| 1jsw:D | a.127.1.1 | 1qk2:A | c.6.1.1 |
| 1v4k:A | a.128.1.1 | 1c81:A | c.60.1.4 |
| 1csm:B | a.130.1.2 | 1mzv:A | c.61.1.1 |
| 1dvg:B | a.132.1.1 | 1grc:B | c.65.1.1 |
| 1mkv:A | a.133.1.2 | 1vid:A | c.66.1.1 |
| 1fgj:B | a.138.1.3 | 1bs0:A | c.67.1.4 |
| 1enk:A | a.18.1.1 | 1pzy:D | c.68.1.2 |
| 1mty:E | a.25.1.2 | 1cuw:A | c.69.1.30 |
| 1t8k:A | a.28.1.1 | 1h17:A | c.7.1.1 |
| 1s36:A | a.39.1.5 | 1q8f:A | c.70.1.1 |
| 1e2a:A | a.7.2.1 | 1rg7:A | c.71.1.1 |
| 1bt2:B | a.86.1.2 | 1lio:A | c.72.1.1 |
| 3atj:B | a.93.1.1 | 1u80:B | c.72.3.1 |
| 1p59:A | a.96.1.1 | 1b7b:B | c.73.1.1 |
| 1dqi:D | b.1.13.1 | 1k0w:E | c.74.1.1 |
| 1l6p:A | b.1.17.1 | 1n2l:A | c.76.1.2 |
| 1jk9:A | b.1.8.1 | 5icd:A | c.77.1.1 |
| 1e44:B | b.101.1.1 | 1tzk:C | c.79.1.1 |
| 1ajo:A | b.29.1.2 | 1tzc:A | c.80.1.1 |
| 3pce:P | b.3.6.1 | 1nzx:B | c.82.1.1 |
| 1rie:A | b.33.1.1 | 13pk:C | c.86.1.1 |
| 1ugs:B | b.34.4.4 | 1ftq:A | c.87.1.4 |
| 1joq:A | b.40.1.1 | 1hg0:D | c.88.1.1 |
| 117e:A | b.40.5.1 | 1pfk:B | c.89.1.1 |
| 1jnw:A | b.45.1.1 | 1hrk:A | c.92.1.1 |
| 1tgs:Z | b.47.1.2 | 3thi:A | c.94.1.1 |
| 1apw:E | b.50.1.2 | 1tee:A | c.95.1.2 |
| 1oa9:A | b.52.1.1 | 1uaq:A | c.97.1.2 |
| 1pqe:A | b.52.2.1 | 1a2p:C | d.1.1.2 |
| 1njt:D | b.57.1.1 | 11as:B | d.104.1.1 |
| 1ibz:D | b.6.1.4 | 1n71:C | d.108.1.1 |
| 2rma:C | b.62.1.1 | 1hx3:B | d.113.1.2 |
| 1oyg:A | b.67.2.2 | 1snn:B | d.115.1.2 |
| 1iny:A | b.68.1.1 | 1f4c:B | d.117.1.1 |
| 1c9u:B | b.68.2.1 | 1aro:L | d.118.1.1 |
| 1v04:A | b.68.6.2 | 1til:A | d.122.1.3 |
| 1jof:F | b.69.10.1 | 1dix:A | d.124.1.1 |
| 1ri6:A | b.69.11.1 | 2jdx:A | d.126.1.2 |
| 1h4j:G | b.70.1.1 | 4mat:A | d.127.1.1 |
| 1fsq:B | b.74.1.1 | 1fit:A | d.13.1.1 |
| 1idj:A | b.80.1.2 | 1tae:B | d.142.2.2 |
| 1s80:B | b.81.1.6 | 1bo1:B | d.143.1.2 |
| 1qwr:B | b.82.1.3 | 1m7q:A | d.144.1.7 |
| 1rxg:A | b.82.2.1 | 1d5f:B | d.148.1.1 |
| 1glb:F | b.84.3.1 | 1ugs:A | d.149.1.1 |
| 1sjn:B | b.85.4.1 | 1ftf:B | d.150.1.2 |
| 1b12:C | b.87.1.2 | 1i9z:A | d.151.1.2 |
| 1l1d:A | b.88.1.3 | 1kyi:M | d.153.1.4 |
| 1tpc:2 | c.1.1.1 | 1b65:A | d.154.1.1 |
| 1j4e:A | c.1.10.1 | 1msv:B | d.156.1.1 |
| 1fiy:A | c.1.12.3 | 1znb:B | d.157.1.1 |
| 1n8w:A | c.1.13.1 | 1it6:A | d.159.1.3 |
| 1xya:A | c.1.15.3 | 1erz:A | d.160.1.2 |
| 1rhc:A | c.1.16.3 | 1i7s:C | d.161.1.1 |
| 1ptg:A | c.1.18.2 | 1j4g:C | d.165.1.1 |
| 1mmf:L | c.1.19.3 | 1dma:B | d.166.1.1 |
| 1tqj:B | c.1.2.2 | 1lru:C | d.167.1.1 |
| 1k4h:A | c.1.20.1 | 1ogx:A | d.17.4.3 |
| 1tx0:A | c.1.21.1 | 2nod:A | d.174.1.1 |
| 1r3y:A | c.1.22.1 | 1xdq:B | d.176.1.1 |
| 1b5t:A | c.1.23.1 | 5pah:A | d.178.1.1 |
| 1r30:A | c.1.28.1 | 157l:A | d.2.1.3 |
| 1xi3:A | c.1.3.1 | 1jaj:A | d.218.1.2 |
| 1jue:A | c.1.4.1 | 1fkt:A | d.26.1.1 |
| 1ct5:A | c.1.6.2 | 1pxv:B | d.3.1.1 |
| 1ah4:A | c.1.7.1 | 1lqp:A | d.32.1.2 |
| 1e70:M | c.1.8.4 | 1jep:A | d.36.1.1 |
| 1a4m:A | c.1.9.1 | 1mkb:A | d.38.1.2 |
| 1v7u:B | c.101.1.1 | 1emv:B | d.4.1.1 |
| 1l7m:A | c.108.1.4 | 3rsd:A | d.5.1.1 |
| 1jke:D | c.110.1.1 | 1b0t:A | d.58.1.2 |
| 1iuq:A | c.112.1.1 | 1w2i:A | d.58.10.1 |
| 1obi:A | c.117.1.1 | 1fva:A | d.58.28.1 |
| 1nn4:C | c.121.1.1 | 1wc3:A | d.58.29.1 |
| 1s20:G | c.122.1.1 | 1rao:A | d.58.30.1 |
| 1xa4:A | c.123.1.1 | 1nue:F | d.58.6.1 |
| 1ne7:D | c.124.1.1 | 1jh7:A | d.61.1.1 |
| 1ro7:C | c.130.1.1 | 1d8h:C | d.63.1.1 |
| 1hzd:A | c.14.1.3 | 1q3g:D | d.68.2.2 |
| 1c41:J | c.16.1.1 | 1dch:H | d.74.1.1 |
| 1i4o:B | c.17.1.1 | 1com:E | d.79.1.2 |
| 1okb:A | c.18.1.1 | 4ota:B | d.80.1.1 |
| 1gco:E | c.2.1.2 | 1o86:A | d.92.1.5 |
| 1pp4:A | c.23.10.4 | 1b6z:B | d.96.1.2 |
| 1h0r:A | c.23.13.1 | 1d6m:A | e.10.1.1 |
| 1s2g:C | c.23.14.1 | 1bgw:A | e.11.1.1 |
| 1qdl:B | c.23.16.1 | 1et0:A | e.17.1.1 |
| 1ou0:B | c.23.17.1 | 1frv:D | e.18.1.1 |
| 1h66:D | c.23.5.3 | 1ubj:S | e.19.1.1 |
| 1i9c:A | c.23.6.1 | 1nvb:B | e.22.1.1 |
| 1egh:D | c.24.1.2 | 1lci:A | e.23.1.1 |
| 1jil:A | c.26.1.1 | 1i2w:B | e.3.1.1 |
| 1roz:B | c.31.1.1 | 1si8:A | e.5.1.1 |
| 1nba:B | c.33.1.3 | 1imb:A | e.7.1.1 |
| 1e20:A | c.34.1.1 | 1aro:P | e.8.1.3 |
| 1um9:A | c.36.1.11 | 1c17:L | f.17.1.1 |
| 1ot3:A | c.37.1.1 | 1c17:M | f.18.1.1 |
| 1jha:A | c.39.1.1 | 1m56:A | f.24.1.1 |
| 1chd:A | c.40.1.1 | 1fw2:A | f.4.2.1 |
| 1gt9:2 | c.41.1.2 | 1i78:A | f.4.4.1 |
| 1woi:E | c.42.1.1 | 1mae:L | g.21.1.1 |
| 1q23:A | c.43.1.1 | 1eyf:A | g.48.1.1 |
